# A cultural and gender-based approach to understanding patient adjustment to chronic heart failure

**DOI:** 10.1186/s12955-020-01482-1

**Published:** 2020-07-18

**Authors:** Jelena Surikova, Ada Payne, Karen-Lee Miller, Arian Ravaei, Robert P. Nolan

**Affiliations:** 1 Peter Munk Cardiac Centre, Behavioural Cardiology Research Unit, University Health Network, 585 University Avenue, 6N-618 NU, Toronto, Ontario M5G 2N2 Canada; 2 Cancer Care Ontario, 620 University Ave, Toronto, Ontario M5G 2L7 Canada; 3 Department of Psychiatry and Institute of Medical Sciences, University of Toronto, 27 King’s College Circle, Toronto, Ontario M5S 3H7 Canada

**Keywords:** Chronic heart failure, Quality of life, Cross-cultural, Self-care, Gender

## Abstract

**Background:**

Persons identifying as Black, Chinese, or South Asian make up the largest minority groups in Canada. Individuals with chronic heart failure (CHF) from these groups experience a greater rate of re-hospitalization and poorer quality of life. Although experts agree that culture can shape the experience of CHF, little is known about how patients from these minority populations define a good quality of life with CHF and what barriers they experience when carrying out self-care behaviours. The aim of this qualitative study was to examine cultural and gender-based influences on quality of life in patients with CHF.

**Methods:**

Purposive sampling included 30 patients (67% male), 18 to 75 years of age, who self-identified as Black (*n* = 8), Chinese (*n* = 9), or South Asian (*n* = 6). Caucasians (*n* = 7) were included as a comparison group. Semi-structured interviews (see the online appendix), lasting approximately 60 min, were conducted, which focused on personal understanding of CHF and living with the disease, including impact on lifestyle and quality of life. An inductive qualitative approach with thematic content analysis was used to develop key insights into individual experience of CHF, as well as cultural and gender-based influences on self-care and quality of life. Descriptive statistics were generated from questionnaire responses.

**Results:**

Five key themes emerged from the narrative analysis of participant interviews: (i) CHF as an emergent reality, (ii) quality of life and disruption of lifecourse milestones, (iii) the challenge to accept CHF and re-evaluation of quality of life; (iv) impact on social activities essential to quality of life, and (v) life with CHF as a commitment to culturally tailored self-care. Participants described the unique impact of CHF on their quality of life, including life trajectory milestones such as dating, parenting, and retirement planning, as well as the importance of accepting their diagnosis, and the reframing goals for living well with heart failure. Positive and negative impacts on social relationships were noted, including sexual intimacy and interactions with spouses, other family members, and co-workers.

**Conclusions:**

Study findings highlight important lifespan, cultural, and gender considerations that can inform the improvement of patient care and quality of life for patients and their families.

## Background

Chronic heart failure (CHF) is a progressive syndrome in which the heart is unable to pump oxygenated blood sufficiently to meet metabolic demands during exercise, or at rest. Symptoms of CHF are diverse and can include fatigue, weakness, exercise intolerance, paroxysmal nocturnal dyspnea, orthopnea, and dependent edema [1]. The prevalence of hospitalization in the initial year following diagnosis is approximately 40%, while the 5-year mortality rate is approximately 45% for women and 60% for men [2]. Ethnic minority populations are disproportionately burdened by having the highest risk of developing CHF, and experience poorer outcomes [3].

Self-care for CHF includes adhering to prescribed medications, monitoring symptoms and weight, restricting fluid and sodium intake, following a heart healthy diet, and exercising regularly [4]. Although self-care activities can improve quality of life, adherence remains poor particularly among people of ethnic minorities [5]. There are several factors that may help explain poor self-care adherence among patients from these groups. First, patients of visible minority groups may not have as much information about their CHF and its treatment as compared to Caucasian patients [6]. As such, it can be more difficult for them to follow recommended self-care guidelines for CHF management [6]. Second, barriers to self-care can be ascribed to socio-cultural norms. More specifically, self-care recommendations from health care professionals might conflict with cultural practices of individuals [7]. For example, staying physically active is a commonly recommended CHF self-care behaviour in Western health care, while resting when feeling ill is the norm in other cultures [7]. Additionally, some patients might have trouble reconciling their cultural preferences (e.g., traditional foods that are high in sodium) with heart healthy ones (e.g., salt-restricted diet) [3].

Gender has been studied extensively as a risk factor in CHF, but it has not been examined as something that can contribute to psychosocial outcome in patients with CHF. Although previous research highlighted the importance of assessing health and quality of life perceptions of patients with CHF, the impact of gender differences was not central to their research [8]. As such, it is critical to understand the role that gender plays in recognition, diagnosis, and self-care management of CHF.

The purpose of this study was to gain a greater understanding of patient adjustment to CHF. Our aim was to identify cultural and gender considerations that might present intervention opportunities in order to improve self-care adherence and quality of life among CHF patients.

## Methods

This study was approved by the Research Ethics Board of the University Health Network. Written informed consent for participation was obtained from all participants prior to participation after the explanation of the purpose and procedure of the study. Participants were informed that the interview would be audio-taped and then transcribed for analysis. We used a purposive sampling strategy to recruit individuals who were: (a) diagnosed with mild to moderate CHF (defined as having a New York Heart Association (NYHA) class I to III and documented left ventricular ejection fraction less than 40%), (b) at least 18 years of age, and (c) self-identified as Black, Chinese, South Asian, or Caucasian (last of which was included as comparison group, Table 1). Recruitment was conducted in an outpatient clinic at a tertiary care hospital that serves a culturally and socially diverse population in Ontario, Canada. Eligible participants were identified through medical chart review. English-speaking research personnel (who also spoke Cantonese, Mandarin, Hindi, or Punjabi) approached potential participants and asked if they would participate in an in-person interview following their clinic visit or at an alternate time. Participants were told that the decision to participate in this study would not affect their care and that they could decline to answer any questions in the interview or withdraw from the study at any point. Participants were also told that their contribution to this research project could inform our effort to improve the quality of life for patients with heart failure.

**Table 1:** Background Characteristics of Participants (*n* = 30)

Variables:	Mean or Number	Percentage or (95% Confidence Interval)
Age: years	54.6	(49.4, 59.8)
Sex: % female	10	33.3
Length of Diagnosis (years)	8.3	(6.0, 10.7)
Racial or ethnic background		
Black	8	27
Chinese	9	30
South Asian	6	20
Caucasian	7	23
Years of Education*	15.3	(14.0, 16.6)
Quality of Life**	77.4	(71.5, 83.3)
NYHA Class*		
I	8	28
I-II	1	3
II	7	24
II-III	9	31
III	4	13

* *n* = 29, due to incomplete data

** as measured by the KCCQ

### Data collection and analysis

Interviewees were asked to speak about their life with CHF, including their understanding of the disease and its impact on their lifestyle and quality of life (conceptualized as the ability to do activities that are important in life [9]. Each semi-structured interview lasted approximately 60 min. Trained bilingual interviewers conducted the interviews in English, Cantonese, Mandarin, Hindi, or Punjabi, based on participant’s preference. After the interview, participants completed the Kansas City Cardiomyopathy Questionnaire (KCCQ), which was used as a measure of quality of life [10]. Audiotapes were transcribed in the language in which the interview was conducted. Six were conducted in Mandarin or Cantonese, and therefore, a bilingual research assistant translated these interviews and another independently verified the translation.

We used an inductive qualitative approach with thematic content analysis to develop key insights into individual experience of CHF, as well as cultural and gender-based influences on self-care and quality of life. All interviews were entered into NVivo 11, which assisted with the initial sorting and coding of data and the identification of higher-level content themes. Descriptive statistics were generated from questionnaire responses. Editing of the quotes from participant interviews was only permitted to remove repetitions or speech dysfluencies.

## Results

Background characteristics of participants are summarized in Table 1. The sample was diverse in terms of age, sex, CHF chronicity, and ethnic background. The KCCQ score was consistent with mild to moderate functional limitation that is common with CHF [11].

Five key themes emerged from the narrative analysis of participant interviews. These are noted below.

### Theme 1: CHF as an emergent reality

Participant comments about their lived experience with CHF highlighted the difficulty of recognizing the significance of their symptoms due to the subtle nature of disease progression.

I have [cardiac] sarcoidosis … It’s insidious. You don’t recognize the destruction because it's incremental, and you also tend to get used to it because … you psychologically adjust the way you do things … You are subconsciously shaping what you do based on limitations without even realizing that you are … . *ID 7, [South Asian], Male, 55 years old*

I used to have this shortness of breath, then I thought that, ‘I think I am getting asthma in my old age’ … when [the doctors] did the echo [cardiogram], they realized it was heart disease. I knew it was a cardiac illness, but I thought when you get heart failure you die instantly.... *ID 20, [South Asian], Female, 71 years old*

Participants interpreted their CHF symptoms in different ways as they sought to understand how their CHF condition could be controlled or managed following diagnosis. In some cases, their illness beliefs reflected poor overall comprehension regarding disease course or aetiology, as well as poor adherence to self-care behaviours that led to serious adverse outcomes:

… I did not take my water pill [on a cruise], so the doctor in Norway told me … “heart failure may cause you to die”. I didn’t realize that it was that serious … [Before that] I didn’t pay too much attention. I [had] said, “as long as it’s not ‘heart attack’ but ‘heart failure’ maybe it’s not as serious as heart attack.” *ID 27, [Chinese], Male, 70 years old*

For two year [s] I suffered like you know for this condition but slowly … like, in the beginning it was a little bit okay right, but slowly … I start losing weight, and … I feel more fatigue, and … short of breath stuff like that and my stomach [is] always full with the fluid … I was doing a little bit of exercise too so I thought myself and my family member thought maybe I am doing that exercise that's the reason that I am losing weight, nobody thought it was because of this [heart condition]. *ID 9, [South Asian], Female, 48 years old*

### Theme 2: Quality of life and disruption of lifecourse milestones

Several participants spoke about the significant disruption of CHF on lifecourse milestones, such as dating, marriage, parenthood, and retirement. As a result, participants reported added stress when they needed to prioritize access to medical treatment over pursuing these milestones:

My plan was to … get married, and do what everybody else does. What happened to me is [I’m] in [my] 20’s … and all of a sudden … they've got to put a box [defibrillator] in [me] … And that kind of changes things … I mean, I’m just trying to stay alive, and so dating is *gone* … The girl I was dating at the time, God I feel sorry for her [because] she never heard from me again … I wasn't about to get her involved in that … . *ID 7, [South Asian], Male, 55 years old*

24 hours after delivering my son … I was in heart failure … I work in a health care field so I could understand what heart failure was. [But] what I struggled with in the beginning – having just had my first child – was, *could I be a mother at all to my son*? I wasn’t able to lift him, or hold him, or care for him … I didn’t have much of a relationship with him over the first 3 months, which actually ended up extending to about a year and a half. *ID 17, [Caucasian], Female, 50 years old*

I wanted to retire back home in Jamaica. I’m not surely able to do that [now] based on my [CHF] treatment, my medications, my access to treatment. I’ll have to confine myself to being in Canada, which wasn’t the plan [in my] golden age. *ID 8, [black], Male, 56 years old*

### Theme 3: Impact on social activities essential to quality of life

Participants spoke at length about the significant impact of CHF on their ability to pursue social activities that were essential to their quality of life. These activities represented key relationships with intimate partners, co-workers, and family members.

#### Sexual intimacy

Several male participants identified personal barriers to sexual intimacy. These barriers included the impact of CHF on emotional concerns or fearfulness of their partner/spouse, and their own experience of erectile dysfunction:

Well I think everybody would like to have sex until they die, but my sex life has slowed down quite a bit … My wife says, “Are you crazy? You’re going to get your heart going!” *ID 22, [Caucasian], Male, 76 years old*

Heart failure as a whole is not a barrier in terms of socializing [with friends] but in terms of intimacy, yeah. It's more of a physical thing than anything, and sometimes it’s frustrating. Actually not sometimes, [but] most of the time … [CHF] plays more in your mind, and [my wife is] more conscious of it kind of thing. *ID 6, [Chinese], Male, 55 years old*

#### Relationships with family members

Some participants also described the negative impact of CHF on their relationships with family members due to activity limitations associated with fatigue and endurance and emotional effects following diagnosis. Participants described how CHF had altered activities that had been essential to their social relationships:

My husband is incredibly active...So we can’t go for bike ride together [because] I’d go maybe 10 or 15 kilometers, and he likes to go for 150. Never mind the speed. So we can’t do many sports together … we don’t do activities together, pretty much across the board. … I think that’s probably been the hardest thing that I’ve had with relationships: appearing normal and knowing that I can’t do what everybody else can do, or what I should be able to do if I was a healthy version of me. *ID 17, [Caucasian], Female, 50 years old*

I had a heart attack two weeks after I gave birth … It did take a toll on my mental health. I had to go on antidepressants … anytime [the heart attack] was brought up, I just started crying … My [older elementary school age] kids used to be in all these activities, like after-school programs, and I would take them to the programs. They only go to programs on Saturdays now because I can’t do it … So [my CHF condition] just changed how we live. *ID 13, [black], Female, 37 years old*

#### Reactions by others

Participants often mentioned the existence or anticipation of problematic reactions by others. Concern was noted about overprotection by family members, which was described as an imposing care that undermined their individual autonomy or status within the social network.

I just didn’t want [friends and co-workers] to know. I don’t want people to treat me differently. I don’t want to be the guy who had a heart attack in his twenties, you know... South Asian moms are overprotective in general. And so like it’s like every 15 minutes she wants to remind me that I have a heart condition. *ID 30, [South Asian],Male, 33 years old*

I weigh myself every morning … and if I haven’t … my wife will ask me everyday what I weigh … If I go up one or two pounds my wife is right there with that pill, saying, “You’re taking this”.... She’s … tough … *ID 22, [Caucasian], Male, 76 years old*

My family, they try to [be] extra sensitive to things. So like there will be times, for example, they won’t invite me to an event or something like that, thinking that it’s too late, … [I] would be too tired … , you know that sort of thing, or “it’s too much, we won’t invite her husband to go along for the weekend, the guys’ weekend trip, because, you know, he’s not gonna wanna leave her for the whole weekend”. But for us we say, “let us be the ones to say no” … . Because they are super sensitive, sometimes they forget that sometimes we just want to be normal … . *ID 14, [Caucasian], Female, 33 years old*

In other cases, participants reported reactions by others that improved their relationships. This often followed the failure of significant others to appreciate functional limitations caused by CHF. These affirming reactions were expressed in diverse ways, such as explicit statements of increased appreciation, demonstrations of appropriate care, or chiding that was experienced as positive support.

Now [my husband] has realized, “she’s literally knocked [at] the doorsteps of death and she’s come back,” and so it has made us really close. He’s very helpful, and you know in that sense he’s taking more care of me. Earlier [he took me] for granted. *ID 5, [South Asian], Female, 35 years old*

I think if anything [CHF] brought my family closer...it just encouraged us to be more healthy and stuff like that together, so yeah it just brought us together a little bit. *ID 4,*[black],*Male, 19 years old*

### Theme 4: The challenge to accept CHF and the re-evaluation of quality of life

Participants provided emotionally charged descriptions of their experience during the immediate aftermath of being diagnosed with CHF. These descriptions reflected the idea of existential threat, which was expressed using terms such as “emotional”, “frightening”, “scared”, and “bad memories.” Themes that were dominant in their comments included the perception of unfairness, fatalism, and denial:

I was in denial [about CHF] more than a year … You think one day they're going to say, “we made a mistake, and you're going to be completely fine.” *ID 5, [South Asian], Female, 35 years old*

It’s like … dealing with a bully: I would prefer not to deal with the bully, and if I could get the bully out of the way, gladly I would...But if I can't, then I have to deal with it. *ID 7, [South Asian], Male, 55 years old*

Many participants reported periods of transition in their life as they learned to cope with the initial onset of CHF, and then with acute medical crises after having this condition for several years. Acceptance of CHF and its impact on life was characterized by expressions of resolution. Participants reported that they established a way to continue with daily activities or to pursue life goals that were meaningful, while living with CHF and the associated functional limitations. This resolution was marked by a reduction in worrying about their longevity, and by appreciating valued activities within the limitations of their “here and now” experience:

When they told me the first time that I might need a new heart, [it] really knocked me for a six [indicates a feeling of devastating surprise] because I thought, “what [are] my chances of getting a heart?” … . But you get to the realization that you can’t worry about that. You can’t do anything about [the transplant waitlist], you know...So we decided that I would just carry on living my life, the best way I knew how. *ID 20, [South Asian], Female, 68 years old*

The first couple of years [after a heart attack], you are certainly more aware of coming to the anniversary of when it happened, and what you were doing [at the time]. So I think for me, I just had to get over that hurdle, and not let it control me, and keep reminding myself that I survived the heart attack, and I’m here to tell the story, and move on and continue to enjoy life … . I don’t let it spoil my life. *ID 11, [Caucasian], Male, 57 years old*

Many participants reported that CHF-related limitations were experienced as a ‘new normal’ that reflected their effort to acknowledge and accept changes in their life that were profound in nature. This active acceptance appeared to diminish anxiety or distress, as participants reinterpreted the ever-present possibility of premature death:

What does [CHF] mean to me? It’s not being able to do everything you were able to do before I was diagnosed. I’ve had it since I was … 40, so it has been 13 years. So that statement kind of loses what it means because after 13 years your life completely changes. And what is abnormal to other people is [now] normal to you*. ID 1, [Caucasian], Male, 53 years old*

You live every day as though it could be your last … I certainly know that in a blink of an eye things can change and go from being completely normal - even though your normal is different than everybody else’s, it’s still your normal - and then, you know, within seconds you can be on your way in an ambulance to the hospital. So you certainly appreciate the little things. You don’t wait for birthdays or Christmas to celebrate. *ID 14, [Caucasian], Female, 33 years old*

### Theme 5: Life with CHF as commitment to culturally tailored self-care

Many participants expressed their acceptance of life with CHF through their commitment to self-care behaviours. However, these behaviours were often passed down by family and friends and heavily shaped by traditional health beliefs and food therapy philosophies. They believed that these behaviours would not cause physical harm, even though they are not typically recommended by Western medical doctors.

My wife said, “eat red stuff”: strawberry, red pepper, raspberry, just red. … whatever vegetable is red, has red components, is good for your heart. *ID 27, [Chinese], Male, 70 years old*

Lately, I have been reading some books recommended by my friends, regarding unblocking blood vessels...boiling garlic, ginger, apple vinegar together, use a cup’s worth of the ‘juice’ after it is boiled, boiling the juices from these 3 things...add honey, … keep in the fridge, drink 2 tablespoons in the morning after waking up. I have been drinking this for a month. I tried this, doesn’t hurt, you can’t die from drinking this*. ID 10, [Chinese], Male, 60 years old*

In some cases, participants found it difficult to reconcile their need to follow heart healthy lifestyle guidelines and maintain culturally salient behaviors that are tied to their cultural identity. This was particularly evident in managing the dietary intake of traditional foods that are high in sodium, such as soy sauce or ham choy (Chinese/Taiwanese), achaar (South Asian), or waakye or jerk-spiced meat (Jamaican):

I am [of] Hakka background so a lot of [meals include] preserved vegetables [which involve] salt frying. How do you avoid that? … For most people with my cultural background, you become more Canadianized … because you have to go with food groups [the dieticians] know. Like celery sticks. For us, we don't eat it raw … whereas every time [dieticians] talk about celery sticks, they talk about dipping sauces and so forth … . *ID 6, [Chinese], Male, 55 years old*

… I would have preferred if [dietitians in the cardiac clinic] could be more specific about our diet because I used to eat a lot of pickle “achaar”, as you know [laughs] and I couldn't survive a single day without having achaar because I am fan of spicy food. But one thing here they did tell me is increase the spice level and reduce the sodium, so that I have started doing. … I really want to go to zero sodium. I don’t know if I can do it because as Indians … for everything we have salt. But we can just try our best. *ID 5, [South Asian], Female, 35 years old*

## Discussion

A patient’s understanding of self-care for CHF is shaped by conventional dominant cultural guidelines by heart health professionals, individual ethnocultural values and beliefs about health, individual competencies for assimilating self-care information, and the individual’s established lifestyle habits and the broader practices of their family or social networks [12]. This qualitative study explored a diverse group of CHF patients and their self-care behaviours within the context of their lived experiences of culture, gender and life trajectory. Close attention was paid to gaining a greater understanding of the impact of CHF on quality of life across the lifespan, and on individual cultural influences on established health care practitioner recommendations.

The experience of CHF as an emergent reality was a major issue observed in the interviews (Theme 1). Our participants described the progressive limitations on their functional ability and psychosocial activity that resulted from CHF. Successful or adaptive adjustment to CHF was reflected in comments about acceptance of their new lifestyle as a “new normal”. This has been reported previously for elderly CHF patients, nevertheless we observed this phenomenon for younger patients as well [13]. In addition, previous research has reported that active coping and acceptance of functional limitations are associated with reduced symptoms of depression and increased quality of life [14, 15]. Narrative data from Theme 4 of our study suggests an important connection. Participant reports indicated that the acceptance of CHF as part of one’s “new normal” may be achieved *through* active coping. To paraphrase from Theme 4, our participants reported that they learned to pursue quality of life by intentionally engaging in daily activities that were within the range of their functional abilities, which made their “new normal” experiences acceptable. This insight leads to an important question for further research: what are the prototypical categories of daily activities through which CHF patients pursue quality of life?

Moreover, our study is distinguished from most qualitative studies in this area that focused on older individuals, due to the prevalence of CHF among persons above 65 years of age [16, 17]. By including younger individuals we were able to explore the impact of CHF on life trajectory milestones such as dating, parenting, and retirement planning, as illustrated in narrative Themes 2 and 3, that we may have otherwise missed. Standardized health-related quality of life instruments have been criticized for neglecting key content areas associated with relationships and parenthood [18]. Our findings highlight the importance that participants attribute to their pursuit of quality of life in areas that are often overlooked in standardized questionnaires, such as sexual intimacy, post-partum bonding and functioning, and retirement planning.

Cross-cultural considerations figured prominently in this study. In Canada, persons identifying as Black, Chinese, or South Asian are the largest visible minority groups [19]. CHF patients from these backgrounds have been observed to experience diminished quality of life [5] and a greater rate of re-hospitalization [20]. This disparity may be complicated by the potential for incongruence between conventional self-care guidelines and the cultural beliefs and practices of CHF patients. This was exemplified by the knowledge and habits around food selection that are shaped by cultural preferences (e.g., the popularity of soy sauce or pickled vegetables) and by beliefs about cardioprotective effects of traditional foods (e.g., “red foods”). Findings from Theme 5 of our present study underscore how an approach to self-care education for CHF that reflects traditional health beliefs and food therapy philosophy can make these materials more culturally relevant, and thus more acceptable to these populations. Several small trials have demonstrated an ability to achieve this goal, as shown by reduced hospitalizations among CHF patients of an ethnic minority group following a multicomponent care management program [21]. The present study highlights the importance for further research to develop and test culturally sensitive interventions that could help facilitate self-care and improve quality of life among visible minorities living with CHF.

One notable difference in the interviews with male and female participants concerned sexual health and intimacy. During the interviews several male participants spontaneously raised concerns related to sexual functioning, while others openly, and without prompting, discussed sexual health or activity within heterosexual relationships. In contrast, female participants did not raise sexual health or sexual activity concerns with interviewers. Instead, the physical impact of CHF on birthing and post-partum experiences, mothering, and the ability to carry out family responsibilities, were at the forefront of women’s concerns. It is unknown whether the lack of mention of female sexual health or sexual dysfunction is related to participants’ personal absence of concern. However, this appears unlikely. Earlier studies have identified that up to 50% of women with cardiovascular disorders note sexual health concerns [22, 23]. Instead, the lack of mention may be due to the low representation of women in this study (33%), which is a clear limitation, and should be redressed in future studies. It is also possible that because interviews were conducted in a hospital setting, this may have led female participants to perceive interviewers in a health provider role, more than in other settings. Previously, women with CHF reported significant discomfort in raising sexual functioning issues with health care providers [22, 23]. Finally, it is possible that absence of discussion on topics related to sexual functioning and dysfunction by women with CHF may reflect reticence to specifically introduce sensitive topics without first being asked by interviewers. Our interviews were open-ended with a general focus on quality of life with CHF. We did not specifically ask about sexual health, and instead allowed participants to raise issues of their own choosing. This approach may have inhibited women or gay men, from speaking about sexual health issues in the interviews. Sexual health in the context of quality of life for women as well as for LGBTQI persons with CHF is an area that should be further explored.

In general, study participants appeared to speak openly about a range of psychosocial issues associated with living with, and adapting to CHF across the lifecourse and within the context of cultural considerations. This was likely facilitated as interviews were conducted in participant’s original language, or their language of choice, which is in contrast to conventional discussions on CHF that are often not conducted in the patient’s mother tongue and which focus on symptom management and options for treatment [24].

## Conclusion

To date, little is known about the activities that CHF patients identify as inherent to a good quality of life with CHF, and the barriers they face when carrying out heart health self-care behaviours, particularly within the context of ethnocultural practices. Our study identified the impact of CHF on life trajectory milestones such as dating, sexual relations, post-partum and parenting, and retirement planning, which is rarely illuminated given the focus on older individuals in CHF research. For individuals from minority ethnocultural backgrounds, self-care often involved culturally-tailored health practices and, at times, uneasy lifestyle and foodstuff compromises in order to adhere to Western health care recommendations.

## Supplementary information

**Additional file 1.**

## Data Availability

Data presented in this study are available from the corresponding author pending approval of the Research Ethics Boards of our host institutions and on reasonable request received from qualified researchers trained in human subject confidentiality protocols.
