# Supplementary material for: A cultural and gender-based approach to understanding patient adjustment to chronic heart failure
Source: Health Qual Life Outcomes. 2020 Jul 18;18:238. doi: 10.1186/s12955-020-01482-1 (PMC7368735; doi:10.1186/s12955-020-01482-1)
Supplement: Supplementary file 1 — Additional file 1. [file 12955_2020_1482_MOESM1_ESM.docx]

**Quality of Life and Unmet Self-Care Information Needs of Chronic Heart Failure Patients: A Cross-Cultural Needs Assessment**

**Interview guide**

** Use probes if the subject did not discuss the specific areas in their response to the corresponding larger question.*

1. How did you find out that you have heart failure?

*Probes:*

- *When did you find out?*
- *What happened before you got you diagnosed?*
- *What treatment did you receive?*
- ***If immigrant & if HF diagnosed before he/she came to Canada:*** *[Clarify the backstory between what happened in home country and in Canada]*
  - *Which part of your medical care happened in [home country]?*
  - *What happened to your medical care (related to HF) after you have arrived in Canada?*

1. When you were told that you have heart failure, what does that mean to you?

*Probes:*

- *What do you know about your heart failure?*
- *Where (or who) did these ideas about heart failure come from?*

3. What is it like for you living with this condition?

*Probes:*

- *How has heart failure affected…*
  - *… your ability to do the things that are important to you?*
  - *… your ability to do the activities that you enjoy?*
  - *… your relationships?*
  - *… your goals in life?*
  - *… your spiritual or religious life?*
  - *… your mental health? (if you are feeling or thinking more positively or negatively)*

4. Can you please tell me more how have you cope with your illness?

*Probes:*

- *What are the major challenges you face?*
- *Is there anything you want to do but can’t do now?*
- *What is holding you back?*
- *What has encouraged you to continue with your life?*
- **if immigrant**: *What was it like for you to deal with living to a new country and managing your heart failure at the same time?*

5. Your health care team has likely been talking to you about **what you can do in order to help your heart health**. Can you tell me what you have heard (or understand what has been told to you?)

*Probe:*

- *Diet? Exercise? Taking medication? Weigh yourself? Limiting salt and fluids? Smoke-free living? Avoid alcohol?*

6. What types of information have you received from your doctor about these heart-healthy lifestyle behaviors?

*Probes:*

- *Format: Pamphlets? Website?*

7. With the lifestyle information given by your doctor, how prepared were you (or are you) to deal with your heart failure?

*Probe:*

- *Is there any lifestyle information that you did not get but would like to have? e.g., diet? Exercise? [Any of the self-care behaviours listed above]*

7. How have these heart-health information received made a difference in your day-to-day life?

***If yes:*** *What differences have they made?*

***If no:*** *What were some of the challenges? barriers?*

*Probes:*

- *Language problems? Not relevant to my life? Not in charge of that part of my life?*

9. Is there anything else you would like to add?

**Summarize participant’s responses:**

Before we finish, I would like to summarize what we have spoken about today. *[Proceed with summary]*

- Helpful knowledge on self-care
- Unhelpful knowledge on self-care
- Helpful self-care practices
- Barriers to self-care practices
